# 1818. Characteristics Associated with Mortality in Patients with Gram-negative Bacteremia in Intensive Care Units

**DOI:** 10.1093/ofid/ofac492.1448

**Published:** 2022-12-15

**Authors:** Alaina Shukdinas, Alissa Werzen, Shereef N Ali, Nikunj M Vyas

**Affiliations:** Jefferson Health, Williamstown, New Jersey; Jefferson Health New Jersey, Voorhees, New Jersey; Jefferson Health New Jersey, Voorhees, New Jersey; Jefferson Health New Jersey, Voorhees, New Jersey

## Abstract

**Background:**

Gram negative rod (GNR) bloodstream infections in patients requiring ICU level of care are associated with high risk of mortality. Very limited data is available on specific risk factors that are associated with this high mortality. The purpose of this study was to identify characteristics associated with mortality in ICU patients who were diagnosed with GNR bacteremia.

**Figure:**
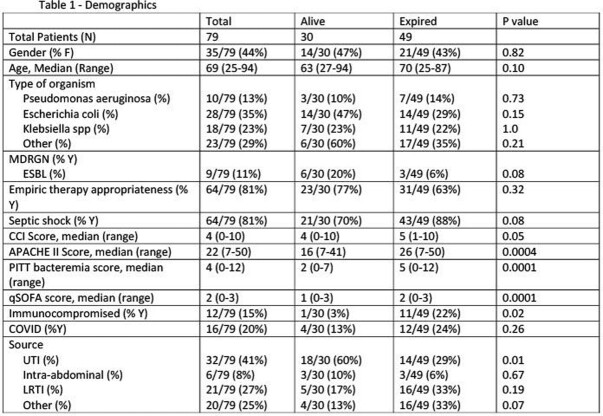

**Methods:**

This was an IRB approved retrospective chart review that was conducted during November 2019 to November 2021 at a three hospital health system in NJ. Patients were included if they required ICU level of care, age ≥ 18 years old and had documented gram negative bacteremia. Patients were excluded if they had metastatic cancer, left against medical advice, or the blood culture was determined to be a contaminant. The primary endpoint of the study was to evaluate the association of characteristics and risk factors with all-cause inpatient mortality (ACIM) in patients with GNR bacteremia requiring ICU level of care. Secondary endpoints included impact of time to appropriate therapy and time to microbiologic cure on ACIM.

**Results:**

There was a total of 79 patients who met inclusion criteria. The median age of patients was 69 years, 44% of the patients were female, and 81% of patients were in septic shock upon presentation to the ICU. The most common source of GNR bacteremia was urinary tract, followed by lower respiratory tract. The most common causative organism of bacteremia was *E.coli,* causing 25% of the cases. When evaluating primary endpoint, higher CCI (OR = 1.79, 95% CI [1.09-2.94]), and Pitt Bacteremia (OR = 2.1, 95% CI [1.32-3.35]) scores were independently associated with an increased risk of mortality in patients with gram-negative bacteremia in the ICU setting. Microbiologic cure was associated with a lower risk of mortality (OR=0.02, 95% CI [0.002-0.2]). Longer time to appropriate therapy and microbiologic cure were not associated with increased ACIM.

Results

**Figure:**
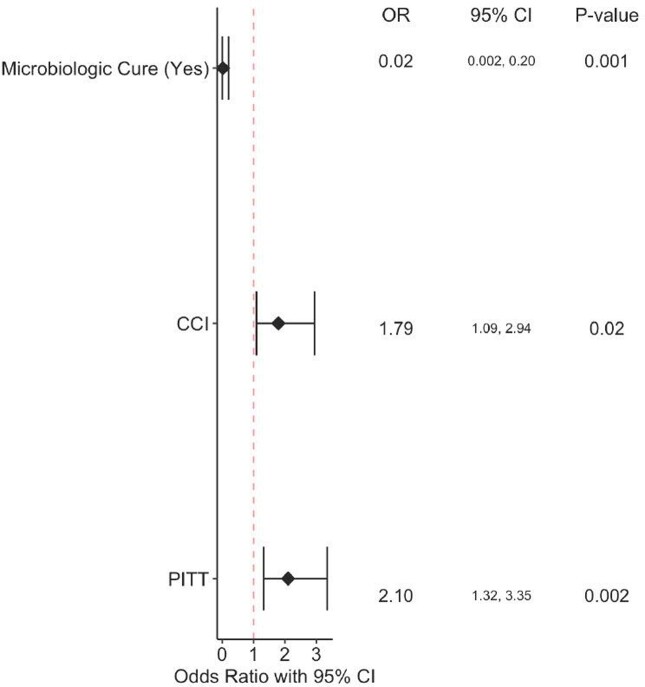

**Figure:**
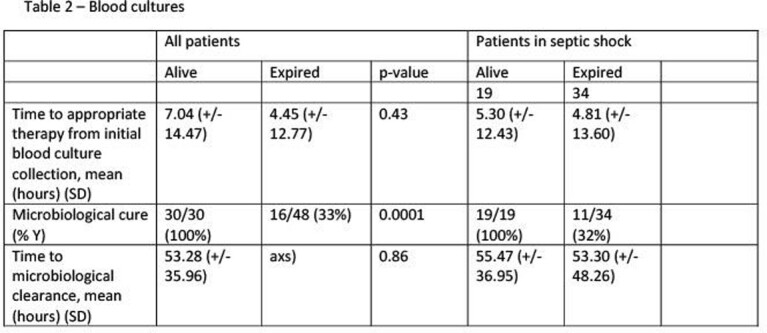

Enterobacterales Breakdown

**Figure:**
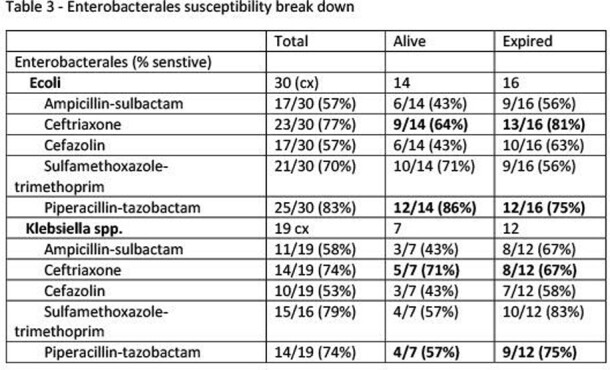

**Conclusion:**

Patients admitted with GNR bacteremia requiring ICU level of care with higher CCI and Pitt Bacteremia scores have higher likelihood of suffering from ACIM. Microbiologic cure was independently associated with lower risk of ACIM. In a subset of patients, longer time to appropriate therapy and microbiologic cure was not associated with increased ACIM.

**Disclosures:**

**All Authors**: No reported disclosures.

